# Trace Metal Inventories and Lead Isotopic Composition Chronicle a Forest Fire’s Remobilization of Industrial Contaminants Deposited in the Angeles National Forest

**DOI:** 10.1371/journal.pone.0107835

**Published:** 2014-09-26

**Authors:** Kingsley O. Odigie, A. Russell Flegal

**Affiliations:** WIGS Laboratory, Environmental Toxicology, University of California Santa Cruz, Santa Cruz, California, United States of America; DOE Pacific Northwest National Laboratory, United States of America

## Abstract

The amounts of labile trace metals: [Co] (3 to 11 µg g^−1^), [Cu] (15 to 69 µg g^−1^), [Ni] (6 to 15 µg g^−1^), [Pb] (7 to 42 µg g^−1^), and [Zn] (65 to 500 µg g^−1^) in ash collected from the 2012 Williams Fire in Los Angeles, California attest to the role of fires in remobilizing industrial metals deposited in forests. These remobilized trace metals may be dispersed by winds, increasing human exposures, and they may be deposited in water bodies, increasing exposures in aquatic ecosystems. Correlations between the concentrations of these trace metals, normalized to Fe, in ash from the fire suggest that Co, Cu, and Ni in most of those samples were predominantly from natural sources, whereas Pb and Zn were enriched in some ash samples. The predominantly anthropogenic source of excess Pb in the ash was further demonstrated by its isotopic ratios (*^208^Pb/^207^Pb: ^206^Pb/^207^Pb*) that fell between those of natural Pb and leaded gasoline sold in California during the previous century. These analyses substantiate current human and environmental health concerns with the pyrogenic remobilization of toxic metals, which are compounded by projections of increases in the intensity and frequency of wildfires associated with climate change.

## Introduction

### Historic Emissions of Trace Metals

Extensive anthropogenic emissions of environmentally persistent contaminants have substantially altered the natural biogeochemical cycles of some trace metals in the biosphere over the past century [1]–[3]. For example, ∼90% and 75% of atmospheric lead (Pb) in California and the world, respectively, during the second half of the previous century were attributed to anthropogenic sources, predominantly the combustion of leaded gasoline [1].

These industrial emissions extensively contaminated aerosols and sediments in Southern California, where enormous amounts of leaded gasoline were combusted during the previous century. For example, ∼8.9% (412,300 metric tons) of the estimated 4,639,000 metric tons of Pb additives used in gasoline in the US between 1950 and 1982 was in California [4]. Subsequently, Pb concentrations in surface soil in Los Angeles Metropolitan area increased from 16±0.5 µg/g (mean ± SD) between 1919 and 1933 to 79±23 µg/g between 1967 and 1970 [5]; and the flux of Pb to sediments in the coastal San Pedro Basin, which abuts the Los Angeles Metropolitan area, increased by ∼7-fold, compared to the natural rate, following the introduction of alkyl-lead gasoline additives in Southern California [6]. Similar temporal increases in fluxes of Pb in age-dated sediments that were attributed to the use of leaded gasoline have been documented in adjacent areas both north and south of Southern California, albeit at lower levels [7], [8]. Substantial temporal increases in the fluxes of other trace metals, e.g., zinc (Zn), associated with anthropogenic inputs have also been measured in Southern California [9].

However, efforts to reduce industrial metal contamination, especially Pb contamination, over the past half century have been quite successful in much of the world, notably the US and specifically Los Angeles. For example, the burden of emissions from the past combustion of leaded gasoline on humans is shown by the relatively high average blood Pb levels (PbB) of children (ages 1–5 years) in the US, which declined from 15 µg/dL in 1976–1980 to 3.6 µg/dL in 1988–1991, during the phase-out and subsequent elimination of leaded gasoline in the US, and then to the current level of 1.3 µg/dL [10], [11]. Despite these marked improvements, the current average PbB level in the US is still about 80-fold greater than the estimated natural PbB (0.016 µg/dL) level in humans [12] and health impairments are now being reported at Pb levels that were previously considered harmless [11], [13]. Similarly, sublethal toxicities of other trace metals (e.g., Cu and Zn) continue to be documented at increasingly lower levels [14].

Moreover, the legacy of historic anthropogenic Pb emissions is evident in terrestrial and aquatic sediments in California and elsewhere [5], [7]. Ten years after Pb was phased out from gasoline in California, concentrations of Pb (77±19 µg/g) in soil in Pasadena, California were still 6-fold greater than the natural (baseline) Pb level (∼12.5 µg/g) of crustal rock [5]. This persistence of industrial Pb deposits was summarized by Steding et al. [15] and Dunlap et al. [1], who predicted that it could take decades to centuries to flush leaded gasoline depositions from surface sediments in California’s Central Valley to San Francisco Bay. Similarly, Semlali et al. [16], based on a study in France, estimated that it would take at least 700 years to reduce the amount of anthropogenic Pb accumulated in soil by 50%.

### Remobilization of Trace Metals by Forest Fires

Wildfires remobilize legacy trace metal contaminants deposited in grasslands and forests. Metals are sequestered in sediment, soil organic matter, and vegetation in forests, where they are relatively immobile [17]–[19]. However, the combustion of vegetation and soil organic matter releases these trace metals in more labile forms [17], [20]. In addition, forest fires increase erosion rates by as much as 100-fold and facilitate the rapid transport of remobilized trace metals to draining water bodies, raising contamination levels in aquatic ecosystems [21], [22].

While there have been several investigations of the remobilization of mercury by forest fires [23], [24], there have been few similar studies on the remobilization of Pb and other trace metals (e.g., Co and Zn). The latter include a recent study in the relatively unpopulated and relatively pristine Santa Barbara area north of Los Angeles [25] and a similar study in Australia [26]. In contrast to those studies, this study was conducted in the Angeles National Forest, which is adjacent to the Los Angeles Metropolitan area, which has a population of ∼17 million [27], has many large sources of industrial contaminants [28]–[30], and ranks as one of the nation’s most contaminated metropolitan areas [31]. Moreover, “car is king” in Los Angeles, where leaded gasoline emissions were the dominant source of atmospheric Pb during the previous century and resuspension of that legacy pollution continues to be the principal component of atmospheric Pb today, as documented in mass balance study of atmospheric Pb contamination in the Los Angeles Air Basin [5]. Therefore, we hypothesized that forest fires within that basin would remobilize relatively large amounts of industrial Pb and other industrial metals, and that remobilization would add to the already large amounts of legacy gasoline Pb deposits that now constitute the major source of atmospheric Pb in Los Angeles.

## Materials and Methods

### Samples Collection Sites

**Figure 1:**
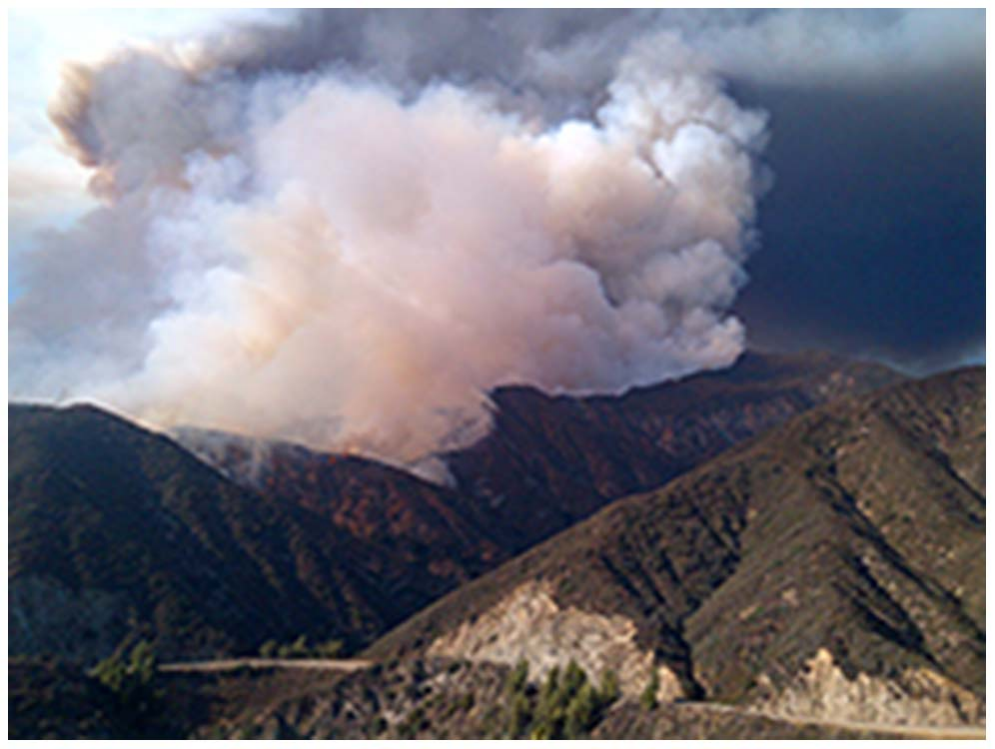
Photograph of the 2012 Williams Fire in Angeles National Forest, California showing the relatively remote and mountainous terrain where the fire occurred. Photo by Freddie W. Duncan, US Forest Service.

**Figure 2:**
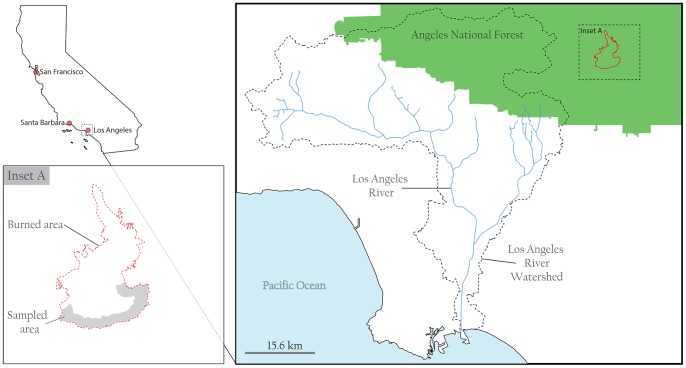
Map showing approximate samples collection sites within the boundary of the 2012 Williams Fire in Angeles National Forest, California.

The Angeles National Forest, which covers ∼ 283,280 hectares (700,000 acres), abuts the Los Angeles Metropolitan Area [32]. The September 2012 Williams Fire scorched about 1,696 hectares (4,192 acres) of land in Angeles National Forest from approximately September 2^nd^ to 13^th^ (Fig. 1). The fire occurred in a very steep (∼ 30–80% slope) area of the forest that is covered with medium to heavy brush. The fuels, mostly chaparral, brush, and mixed conifers, were approximately 15 to 20 years old [33]. Fourteen ash and 6 soil (from unburned patches) samples were collected from different accessible locations within the burned perimeter of the fire site (Figs. 1 and 2) using established trace metal clean techniques [25]. The samples were then transported in plastic containers to the University of California, Santa Cruz for processing. The collection location (GPS coordinates) for each sample is presented in Table S1 in File S1. No specific permissions were required to access and collect samples from the fire site, and the sampling did not involve interactions with endangered or protected species.

### Analytical Methods

Trace metal or ultra-pure (2x sub-boiling quartz distilled) grade reagents and high purity (18.2 MΩ cm) water (Milli-Q) were used for cleaning labware and processing samples. Fourteen ash and 6 soil samples, 3 procedural blanks, and triplicates of 3 National Institute of Standards and Technology (NIST) Standard Reference Materials (SRMs), SRM 1547 (peach leaves), SRM 2709 (San Joaquin soil), and SRM 1633b (coal fly ash), were processed concurrently in a Class-100 HEPA-filtered trace metal clean laboratory. Three ash samples (CWA-1, CWA-8, and CWA-14) and 2 soil samples (CWS-1 and CWS-6) were processed in triplicate.

A concentrated aqua regia (HNO_3_: HCl, 1∶3 v/v) digestion was used to extract the acid-leachable (labile) trace (Co, Cu, Ni, Pb, and Zn) and major (Al and Fe) metals in the samples and reference materials [34], [35] using established methods [8]. These metals were selected to represent a suite of inorganic contaminants emitted from anthropogenic sources at different – from relatively low (e.g., Ni) to high (e.g., Pb) – levels in Los Angeles [29], [36], [37]. Briefly, approximately 0.3 to 0.5 g of ash, soil or reference material was transferred to a clean Teflon digestion vial, dried overnight at 65°C, refluxed in hot (∼130°C) aqua regia for about 6 hours, dried, weighed and redissolved in 10 mL of 1M ultrapure HNO_3_.

The digests were analyzed for trace metal (Co, Cu, Ni, Pb, and Zn) concentrations with a Finnigan ELEMENT 2 high resolution inductively coupled plasma – mass spectrometer (HR ICP-MS), and for Al and Fe concentrations with a PerkinElmer Optima 4300 DV inductively coupled plasma – optical emission spectrometer (ICP-OES). The concentrations of Fe were used to compute the enrichment factors of trace metals in the samples. The samples were then analyzed for stable lead isotopic composition after optimizing their concentrations with a Finnigan ELEMENT 2 HR ICP-MS. Internal standards were used to correct for instrumental drifts during analysis, and NIST SRM 981 (common lead) was used as standard to correct for instrumental fractionation of lead isotopic composition. The detection limit (3 x standard deviation of procedural blanks), reproducibility, and recoveries of each analyte are presented in Tables 1–4. The recoveries of metals in the NIST SRM 1547 (peach leaves) are assumed to be most representative of the metals in the ash samples since they were derived predominantly from vegetation and soil organic matter.

**Table 1:** Detection limits of analytes.

	Detection Limit (3 x SD, Standard Deviation of 3 blanks)						
	(ng/g)					(µg/g)	
Element	[Co]	[Cu]	[Ni]	[Pb]	[Zn]	[Al]	[Fe]
Detection Limit	0.002	0.010	0.002	0.008	0.148	0.015	0.035

**Table 2:** Digestion recoveries (concentrations) for elements in standard reference materials (SRMs).

	% Recovery (Mean ± SD, n = 3)						
SRM	Co	Cu	Ni	Pb	Zn	Al	Fe
NIST SRM 1547 (Peach Leaves)	82.6±3.0	89.3±0.7	74.5±2.8	104±0.8	98.7±1.2	65.7±5.2	86.7±4.7
NIST SRM 2709 (San Joaquin Soil)	52.0±0.4	49.1±0.6	49.1±0.4	77.5±0.7	53.2±0.7	55.6±1.1	93.7±0.5
NIST SRM 1633b (Coal Fly Ash)	43.7±0.7	38.6±0.5	42.6±0.6	62.5±0.3	41.2±0.0	25.6±0.8	76.7±1.9

**Table 3:** Analytical (instrumental) precision.

	% RSD (n = 5)					% RSD (n = 4)	
SRM	[Co]	[Cu]	[Ni]	[Pb]	[Zn]	[Al]	[Fe]
SRM 1640a	0.9	1.1	0.9	0.5	1.6	–	–
Consistency Standard A	2.3	1.6	2.3	0.2	0.9	0.3	0.5
Consistency Standard B	–	–	–	–	–	0.3	0.6

**Table 4:** Analytical (instrumental) precision for lead isotopic compositions.

	% RSD (n = 5)		
Sample	^208^Pb/^207^Pb	^206^Pb/^207^Pb	^207^Pb/^204^Pb
CWA-2	0.07	0.04	0.09

## Results and Discussion

### Metal Concentrations

The “acid-leachable” concentrations of Al, Co, Cu, Fe, Ni, Pb, and Zn, and lead isotopic ratios (^208^Pb/^207^Pb, ^206^Pb/^207^Pb, and ^207^Pb/^204^Pb) of the ash and soil samples are listed in Table 5. The enrichment factors of the trace metals (Co, Cu, Ni, Pb, and Zn) normalized to Fe are listed in Table S2 in File S1. These concentrations and enrichment factors are considered conservative because they may not include metals volatilized during the fire and those contained in refractory aluminosilicate lattices, which require dissolution with concentrated HF [38], [39].

**Table 5:** Concentrations (µg g^−1^ ) of trace metals: Co, Cu, Ni, Pb, and Zn; concentrations (mg g^−1^) of Al and Fe; and lead isotopic composition of ash (CWA) and soil (CWS) samples collected from the 2012 Williams Fire in Angeles National Forest in Southern California.

Sample ID	Concentrations [mean ± *(standard deviation, SD)*]							Lead Isotopic Ratios [mean ± *(SD)*]		
	[Co] (µg g^−1^)	[Cu] (µg g^−1^)	[Ni] (µg g^−1^)	[Pb] (µg g^−1^)	[Zn] (µg g^−1^)	[Al] (mg g^−1^)	[Fe] (mg g^−1^)	^208^Pb/^207^Pb	^206^Pb/^207^Pb	^207^Pb/^204^Pb
CWA-1*	3.90	15.4	6.77	16.0	216	13.8	10.9	2.454	1.189	15.673
	*(0.05)*	*(0.24)*	*(0.25)*	*(0.04)*	*(2.92)*	*(0.55)*	*(0.29)*	*(0.001)*	*(0.001)*	*(0.004)*
CWA-2	7.54	68.5	10.6	24.0	128	18.4	25.0	2.461	1.185	15.635
CWA-3	3.59	41.3	9.21	42.2	83.0	5.79	6.94	2.438	1.178	15.656
CWA-4	6.76	30.8	10.6	14.8	85.0	18.7	24.4	2.457	1.190	15.633
CWA-5	3.81	38.6	9.72	20.8	65.3	7.86	10.8	2.454	1.192	15.659
CWA-6	8.75	56.0	12.5	27.2	414	13.6	16.2	2.463	1.213	15.684
CWA-7	5.17	65.5	14.3	38.5	203	14.3	13.8	2.451	1.195	15.711
CWA-8*	10.9	58.2	13.3	8.95	374	13.0	17.0	2.457	1.193	15.660
	*(0.74)*	*(3.24)*	*(0.52)*	*(1.02)*	*(15.7)*	*(0.36)*	*(0.55)*	*(0.001)*	*(0.002)*	*(0.011)*
CWA-9	4.32	17.3	10.5	9.68	96.5	14.3	16.2	2.471	1.186	15.662
CWA-10	7.47	26.3	14.6	20.0	73.6	24.4	31.5	2.458	1.187	15.672
CWA-11	3.13	17.0	5.86	8.23	74.2	7.42	8.71	2.450	1.189	15.733
CWA-12	6.05	20.5	11.2	17.3	65.5	18.6	18.1	2.462	1.183	15.669
CWA-13	6.30	56.8	9.17	11.5	503	7.85	10.7	2.472	1.188	15.664
CWA-14*	6.66	43.3	12.9	7.38	66.6	20.7	19.2	2.450	1.189	15.686
	*(0.27)*	*(0.23)*	*(0.15)*	*(0.25)*	*(0.68)*	*(0.53)*	*(0.39)*	*(0.001)*	*(0.001)*	*(0.017)*
CWS-1*	7.31	20.0	6.04	20.7	44.5	29.2	52.4	2.459	1.181	15.635
	*(0.10)*	*(3.89)*	*(0.14)*	*(0.76)*	*(0.46)*	*(2.11)*	*(1.84)*	*(0.002)*	*(0.000)*	*(0.011)*
CWS-2	8.65	19.6	21.1	143	61.2	35.1	46.8	2.461	1.204	15.647
CWS-3	12.5	32.1	16.8	20.3	41.5	32.1	43.5	2.455	1.178	15.614
CWS-4	6.26	25.9	11.8	8.62	32.0	20.7	28.1	2.506	1.196	15.684
CWS-5	8.82	17.0	15.0	10.9	37.3	34.9	40.1	2.491	1.185	15.643
CWS-6*	9.98	20.0	13.1	16.4	44.2	33.5	46.4	2.443	1.160	15.581
	*(0.67)*	*(0.89)*	*(1.21)*	*(1.81)*	*(0.65)*	*(5.00)*	*(1.37)*	*(0.001)*	*(0.006)*	*(0.024)*
**Jesusita Fire ash, Santa Barbara, California (Data Summary, n = 30)**										
Mean, [x-]	4.46	–	22.7	18.8^‡^	151	16.7^‡^	13.9^‡^	2.466^‡^	1.205^‡^	15.635^‡^
Range (Min	1.95	–	8.88	4.29^‡^	41.6	5.70^‡^	5.00^‡^	2.436^‡^	1.161^‡^	15.563^‡^
to	–	–	–	–	–	–	–	–	–	–
Max)	7.23	–	49.1	51.0^‡^	433	36.0^‡^	31.1^‡^	2.481^‡^	1.218^‡^	15.716^‡^

Samples marked with (*) were processed in triplicates and are reported as mean ± (standard deviation, SD). Also listed in the table are the ranges and mean concentrations of metals and lead isotopic compositions of ash collected from the 2009 Jesusita Fire in Santa Barbara, California. Data marked with (‡) were summarized from Odigie and Flegal [25].

Concentrations of Co (3.1 to 11 µg g^−1^), Cu (15 to 69 µg g^−1^), Ni (5.9 to 15 µg g^−1^), Pb (7.4 to 42 µg g^−1^), and Zn (65 to 500 µg g^−1^) in the ash varied substantially. The ranges of these concentrations are consistent with those measured in ash collected from the 2009 Jesusita Forest Fire site in Santa Barbara, California (Table 5). The concentrations are also comparable to concentrations of these metals in ash sourced primarily from the experimental combustion of ponderosa pines in Montana [40]. The relatively high Zn levels potentially originated from anthropogenic sources, which have typically included fossil fuel combustion exhaust, galvanized parts and railings, break lining wear, and tire wear [41], [42].

Variations in the amounts of these remobilized metals presumably reflect variations in baseline concentrations of the metals, anthropogenic input, and levels of volatilization of organic matter during the fire [43], [44]. The concentrations of Co (6.3 to 13 µg g^−1^), Cu (17 to 32 µg g^−1^), Ni (6.0 to 21 µg g^−1^), Pb (8.6 to 140 µg g^−1^), and Zn (32 to 61 µg g^−1^) in the soil samples are also comparable to previously reported values of soils in Southern California [45]–[47]. The concentrations of some metals (e.g., Co and Ni) in the soils approximate their baseline concentrations in Southern California, but the >2-fold increase in Pb concentrations (e.g., 140 µg g^−1^) indicated that the Angeles National Forest site had received substantial Pb inputs from anthropogenic sources.

### Enrichment Factors

The amounts of anthropogenic trace metals in the ash are indicated by the enrichment factors of the metals normalized to Fe: Co (0.9 to 2.5), Cu (0.9 to 6.2), Ni (0.3 to 1.0), Pb (1.9 to 29), and Zn (0.9 to 19) (Table S2 in File S1).


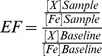
 We calculated the enrichment factor of each metal by normalizing the concentrations of the metal ([X]_Sample_) and iron ([Fe]_Sample_) in the sample to the baseline concentrations of each metal ([X]_Baseline_) and iron ([Fe]_Baseline_) of ^210^Pb-dated preindustrial sediment deposited in San Pedro Basin, which drains parts of the Los Angeles Metropolitan Area. Normalization of trace metals to Fe is considered more appropriate than normalization to Al for samples that were not treated with HF because of the recalcitrance of aluminosilicates as indicated by the difference in the recoveries of these metals in the NIST SRMs (Table 2). Using the enrichment classification proposed by Sutherland [48], the ratios indicate that: Co was not measurably enriched (EF <2) in 10 samples and moderately enriched (EF = 2–5) in 4 samples; Cu was not measurably enriched in 5 samples, moderately enriched in 7 samples, and significantly enriched (EF = 5–20) in 2 samples; Ni was not measurably enriched in all 14 ash samples; Pb was not measurably enriched in 1 sample, moderately enriched in 7 samples, significantly enriched in 5 samples, and very highly enriched (EF = 20–40) in 1 sample; and Zn was not measurably enriched in 4 samples, moderately enriched in 5 samples, and significantly enriched in 5 samples. These industrial enrichments were further indicated by the simple linear correlations between the concentrations of the metals and the concentrations of Fe: [Co] (r = 0.576, p = 0.031, n = 14), [Cu] (r = 0.058, p = 0.844, n = 14), [Ni] (r = 0.612, p = 0.020, n = 14), [Pb] (r = −0.158, p = 0.590, n = 14), and [Zn] (r = −0.221, p = 0.448, n = 14) in the ash. Metals that are primarily from natural origin, e.g., [Al] and [Fe] (r = 0.909, p = 0.000007, n = 14), which are relatively abundant and not easy to contaminate, correlate better than those enriched by anthropogenic inputs [49]. The addition of anthropogenic metal, e.g., Pb, to a system often changes its relative abundance to normalizing major elements as indicated by the correlation of [Pb] and [Fe] above. These varying enrichments are also generally consistent with those of these metals in ash collected from the 2009 Jesusita Fire in Santa Barbara, California [e.g., 25].

### Lead Isotopic Compositions

**Figure 3:**
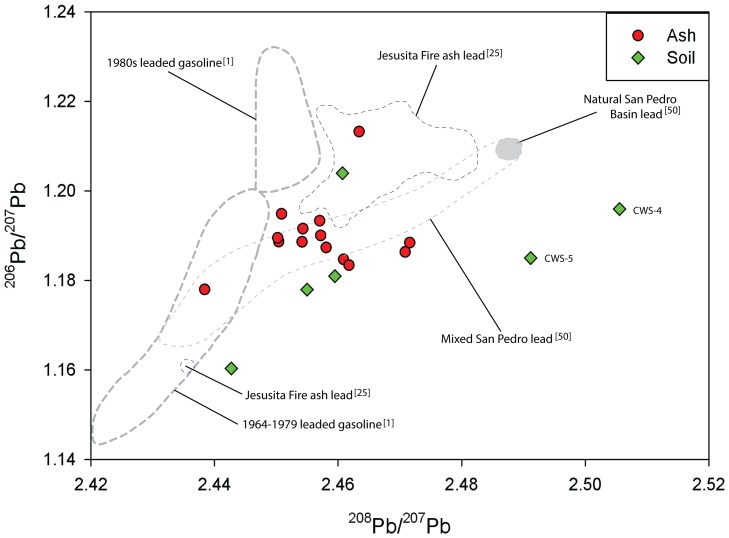
Lead isotopic composition (^208^Pb/^207^Pb: ^206^Pb/^207^Pb) of ash and soil samples collected from the 2012 Williams Fire site in Southern California. The plot also includes the ranges of isotopic composition of alkyl-lead used in California and those of ^210^Pb dated sediment cores in the San Pedro Basin. Also included in the plot are the ranges of Pb isotopic composition of ash collected from the 2009 Jesusita Fire in Santa Barbara, California.

The apparent anthropogenic enrichment of Pb in some of the samples is further demonstrated in Figure 3, which is a plot of the lead isotopic ratios (^208^Pb/^207^Pb:^ 206^Pb/^207^Pb) of the ash and soil samples. The plot also includes the lead isotopic compositions of pre-industrial and contaminated sediment deposited in the San Pedro Basin, which drains parts of the Los Angeles Metropolitan area as previously mentioned, and those of leaded gasoline sold in California during the second half of the last century.

The plot indicates that the Pb in the ash is mainly from anthropogenic sources, predominantly the past combustion of leaded gasoline. The plot also indicates that the portion of anthropogenic Pb from the leaded gasoline used between 1964 to 1979 was substantially larger than that from the 1980s leaded gasoline in the ash, which contrasts the results from the Santa Barbara study that indicate that the predominant source of anthropogenic Pb was the combustion of the 1980s leaded gasoline [25]. The predominantly alkyl-lead in the samples is further corroborated by the isotopic compositions of Pb in most (4) of the soil samples (Fig. 3). Two soil samples (CWS-4 and CWS-5) have distinct Pb isotopic compositions compared to the other samples and are assumed to be representative of mixtures between baseline and anthropogenic Pb or variants of baseline Pb isotopic compositions.

The apparent leaded gasoline signature of Pb in the samples attests to the persistence of industrial Pb deposits in the Los Angeles Basin that is consistent with the results of the mass balance study by Harris and Davidson [5]. It is also consistent with previous reports on the persistence of industrial Pb deposits in California’s Central Valley, which is north of Los Angeles, based on both mass balance and lead isotopic composition analyses [1], [15]. The latter has been principally traced to atmospheric depositions from the combustion of leaded gasoline in California [1].

This attribution is based on the temporal changes in the isotopic compositions of leaded gasoline in California in the previous century. Before 1960, alkyl-lead in the US was primarily manufactured with ores from Australia, Canada, Mexico, and Peru, which had ^206^Pb/^207^Pb ratios of 1.037, 1.064, 1.192, and 1.200, respectively. Then, the proportion of Pb sourced from the Missouri deposits (^206^Pb/^207^Pb = 1.28 to 1.33) increased from 9% in 1962 to 82% in 1976 [50]. This change in the sources of Pb in alkyl-lead was subsequently reflected in the Pb isotopic compositions of aerosols in Southern California. There, the ^206^Pb/^207^Pb ratio of aerosols, which was ∼1.15 before 1967, increased to 1.20 by 1974, and then to 1.23 by 1977 [51].

### Accelerated Transport of Contaminants to Water Bodies and Atmosphere

In addition to atmospheric contamination, wildfires increase the load of contaminants in aquatic ecosystems. This was recently documented in a study [22] that examined the impact of wildfires on contaminant loadings in Southern California – the same area where the Williams Fire occurred. The results from that study show that the mean fluxes of Cu, Pb, Ni, and Zn from burned sites were 110-fold, 740-fold, 82-fold, and 110-fold greater compared to their respective fluxes at similar unburned (control) sites. That study also found that the mean post-fire concentrations of Cu, Pb, Ni, and Zn in the first stormwater collected from an area that was ∼20 km from the closest fire site were each 3 times their pre-fire levels, which they attributed to ash fallout. Similarly, Sabin et al. [52] reported 4-fold, 6-fold, 8-fold, and 13-fold post-fire increases in fluxes of Cu, Zn, Pb, and Ni, respectively, over their mean fluxes in an unburned site in Southern California, which were likewise attributed to forest fires in nearby mountains. Those attributions are consistent with results from a study by Young and Jan [53], who showed that trace metals (Cu, Ni, Pb, and Zn) fallout increased by up to 3-fold in a 10,000 km^2^ unburned area that was impacted by smoke plumes and ash from wildfires in the Angeles National Forest in Southern California.

### Potential Impact of Climate Change

The mobilization of contaminants by wildfires is a concern, especially in the western US where climate change is expected to dramatically expand fire regimes [54]. Recent studies have linked increases in fire intensity, frequency, and burned area to climate change [55]–[57]. For example, wildfire frequency has been positively linked (r = 0.76, p<0.001, n = 34) to regional spring and summer temperatures in western US [54]. The frequency of wildfires and total area burned in western US in the mid-1980s were ∼4 times and ∼7 times, respectively, higher than their mean values from 1970 to 1986, and this interval was marked by a rise in temperature of <0.9°C [54]. Furthermore, the Intergovernmental Panel on Climate Change has projected that global mean surface temperature could increase by up to 4.8°C by the end of this century (2081–2100) relative to 1986–2005, and researchers have predicted a corresponding increase in the frequency and intensity of wildfires [58]–[61]. Consequently, it is hypothesized that the pyrogenic remobilization and volatilization of contaminants from forests and grasslands will increase in response to projected climate change. That expected increase is a concern, especially in forests contaminated with radioactive pollutants and at sites used for nuclear waste storage, as recently highlighted for Chernobyl, Ukraine and Los Alamos National Laboratory, US, respectively [62]–[64]. The results from this and complementary studies provide empirical evidence that wildfires are already remobilizing and volatilizing other contaminants – albeit more pedestrian ones – that constitute a greater global health problem (e.g., Pb).

## Supporting Information

File S1 **Tables S1–S2.** Table S1: Collection locations (coordinates) for ash (CWA) and soil (CWS) samples collected from the 2012 Williams Fire site in the Angeles National Forest in California. Table S2: Enrichment factors (*f*-Fe, normalized to Fe) of trace metals in ash (CWA) samples collected from the 2012 Williams Fire site in the Angeles National Forest in California.
(DOCX)
