# Supplementary material for: Trace Metal Inventories and Lead Isotopic Composition Chronicle a Forest Fire’s Remobilization of Industrial Contaminants Deposited in the Angeles National Forest
Source: PLoS One. 2014 Sep 26;9(9):e107835. doi: 10.1371/journal.pone.0107835 (PMC4178038; doi:10.1371/journal.pone.0107835)
Supplement: File S1 — Tables S1–S2. Table S1: Collection locations (coordinates) for ash (CWA) and soil (CWS) samples collected from the 2012 Williams Fire site in the Angeles National Forest in California. Table S2: Enrichment factors (f-Fe, normalized to Fe) of trace metals in ash (CWA) samples collected from the 2012 Williams Fire site in the Angeles National Forest in California. (DOCX) [file pone.0107835.s001.docx]

Trace Metal Inventories and Lead Isotopic Composition Chronicle a Forest Fire’s Remobilization of Industrial Contaminants Deposited in the Angeles National Forest

Kingsley O. Odigie and A. Russell Flegal

File S1

Table S1: Collection locations (coordinates) for ash (CWA) and soil (CWS) samples collected from the 2012 Williams Fire site in the Angeles National Forest in California.

| Sample ID | Longitude | Latitude | Remarks |
| --- | --- | --- | --- |
| CWA-1 | 117^o^ 48' 40" W | 34^o^ 14' 15" N |  |
| CWA-2 | 117^o^ 48' 36" W | 34^o^ 14' 16" N |  |
| CWA-3 | 117^o^ 48' 31" W | 34^o^ 14' 21" N |  |
| CWA-4 | 117^o^ 48' 01" W | 34^o^ 14' 02" N |  |
| CWA-5 | 117^o^ 47' 16" W | 34^o^ 14' 09" N |  |
| CWA-6 | 117^o^ 47' 15" W | 34^o^ 14' 12" N |  |
| CWA-7 | 117^o^ 47' 17" W | 34^o^ 14' 14" N |  |
| CWA-8 | 117^o^ 46' 13" W | 34^o^ 14' 27" N |  |
| CWA-9 | 117^o^ 45' 53" W | 34^o^ 15' 06" N |  |
| CWA-10 | 117^o^ 46' 05" W | 34^o^ 14' 32" N |  |
| CWA-11 | 117^o^ 48' 28" W | 34^o^ 14' 12" N |  |
| CWA-12 | 117^o^ - ' - " W | 34^o^ - ' - " N | no complete GPS data |
| CWA-13 | 117^o^ 47' 18" W | 34^o^ 14' 09" N |  |
| CWA-14 | 117^o^ 48' 19" W | 34^o^ 14' 11" N |  |
| CWS-1 | 117^o^ 48' 01" W | 34^o^ 14' 03" N |  |
| CWS-2 | 117^o^ 47' 18" W | 34^o^ 14' 08" N |  |
| CWS-3 | 117^o^ 47' 17" W | 34^o^ 14' 10" N |  |
| CWS-4 | 117^o^ 47' 54" W | 34^o^ 14' 01" N |  |
| CWS-5 | 117^o^ 48' 14" W | 34^o^ 14' 06" N |  |
| CWS-6 | 117^o^ 48' 19" W | 34^o^ 14' 11" N |  |

Table S2: Enrichment factors (*f*-Fe, normalized to Fe) of trace metals in ash (CWA) samples collected from the 2012 Williams Fire site in the Angeles National Forest in California.

|  | Enrichment Factors (*f*) | | | | |
| --- | --- | --- | --- | --- | --- |
| Sample ID | *f*-Fe (Co) | *f*-Fe (Cu) | *f*-Fe (Ni) | *f*-Fe (Pb) | *f*-Fe (Zn) |
| CWA-1 | 1.4 | 1.5 | 0.5 | 7.1 | 8.0 |
| CWA-2 | 1.2 | 2.9 | 0.3 | 4.7 | 2.1 |
| CWA-3 | 2.0 | 6.2 | 1.0 | 29 | 4.9 |
| CWA-4 | 1.1 | 1.3 | 0.3 | 2.9 | 1.4 |
| CWA-5 | 1.4 | 3.8 | 0.7 | 9.4 | 2.5 |
| CWA-6 | 2.1 | 3.6 | 0.6 | 8.1 | 10 |
| CWA-7 | 1.4 | 5.0 | 0.8 | 13 | 5.9 |
| CWA-8 | 2.5 | 3.6 | 0.6 | 2.6 | 9.0 |
| CWA-9 | 1.0 | 1.1 | 0.5 | 2.9 | 2.4 |
| CWA-10 | 0.9 | 0.9 | 0.4 | 3.1 | 0.9 |
| CWA-11 | 1.4 | 2.0 | 0.5 | 4.6 | 3.5 |
| CWA-12 | 1.3 | 1.2 | 0.5 | 4.6 | 1.5 |
| CWA-13 | 2.2 | 5.6 | 0.7 | 5.2 | 19 |
| CWA-14 | 1.3 | 2.4 | 0.5 | 1.9 | 1.4 |
| Min | 0.9 | 0.9 | 0.3 | 1.9 | 0.9 |
| Max | 2.5 | 6.2 | 1.0 | 29 | 19 |
